# Opportunities and barriers for food intake in older age – a Norwegian perspective

**DOI:** 10.29219/fnr.v66.8628

**Published:** 2022-11-11

**Authors:** Øydis Ueland, Ida Synnøve Grini, Ine Schillinger, Paula Varela

**Affiliations:** 1 Nofima, Osloveien 1, 1430 Ås, Norway; 2 The Norwegian University of Life Science, Department of Chemistry, Biotechnology and Food Science (KBM), Ås, Norway

**Keywords:** older adults, food consumption, appetite, food and health, food behavior

## Abstract

**Background:**

The ageing processes occur slowly over time and are often not detectable by the individual. Thus, preparing for dietary needs in later years should start at an earlier age than most people realise.

**Objective:**

This study aims at better understanding what characterises food-related practices in active, home-living older adults, in order to identify food-related factors that act as barriers and those that promote healthy ageing.

**Design:**

Three experiments were conducted: First, a web-based quantitative survey to collect information about home-living older adults’ food-related behaviours (67+ years, *N* = 1,005). Second, two focus groups with respondents 67–74 years (*N* = 7) and 75–84 years (*N* = 6) to elicit aspects not adequately covered in the survey. Third, 10 individual interviews to provide in-depth insights.

**Results:**

Two distinct groups were identified in the survey; 67–79 years and 80+ years. The older age group experienced more barriers and restrictions in food intake and food-related behaviours compared to the younger group. Good taste, routines and social settings were important for appetite and food intake.

**Discussion:**

Using a mixed-methods approach proved valuable for extracting information and a better understanding of what impacts on food-related aspects amongst older adults. Strategies for upholding a healthy food intake involve establishing daily routines and meeting arenas where older adults can socialise and eat food together.

**Conclusion:**

This study confirmed that knowledge of older adults’ physical needs, barriers and abilities must be a part in preparation for a healthy diet.

## Popular scientific summary

- As one age, challenges arise that influence older adults’ food intake negatively. In this study, we have investigated food-related practices that characterise Norwegian active, home-living older adults. Insights were collected through a quantitative survey (*N* = 1,005), two focus groups (*N* = 7 and 6, respectively), and 10 in-depth interviews. The results showed that the age group 80+ years experienced more barriers and restrictions in food intake and food-related behaviours compared to the younger group. Good taste, routines and social settings were important for a good appetite and food intake. Strategies for upholding a healthy food intake involve establishing daily routines and meeting arenas where older adults can socialise and eat food together.

*‘I eat because I have to eat. It’s like nothing interests me anymore… I am never hungry, but I eat every third hour when I’m home’*. (Female, 89 years)

*‘Now we can treat ourselves to whatever we want’*. (Female, 85 years)

The world’s ageing population is causing challenges for governments and societies in increasing demands for health care and support (1). This has led governments, non-governmental organisations and international and national institutions to search for knowledge and develop strategies and plans to cope with these needs (2–4). A vision for this work is to achieve more healthy life years for the elderly population. One avenue towards upholding a good and self-reliant life for older people is focusing on adequate food intake (5–8).

The ageing processes occur slowly over time and are often not detectable by the individual. Consequently, necessary changes to the diet due to ageing may not be acknowledged or effectuated if they are not prescribed. Muscle mass starts deteriorating already from the fifth decade (9, 10). In addition to a general decline in muscle mass, Sarcopenia, a muscle deteriorating disease, is common amongst older adults, sometimes occurring already from the 4th decade (9, 11). In this condition, skeletal muscle fibres decline both in size and numbers, which again lead to less muscle strength and physical performance, as well as increased risk of cardiac disease, respiratory disease, frailty and cognitive impairment (9, 11). Advice to mitigate the adverse effects of muscle mass reduction, including Sarcopenia, includes dietary and physical interventions (10, 12). Furthermore, older adults, in particular females, have higher loss of bone mass and prevalence of osteoporosis, which increase risk of falls and fractures (13, 14). An optimised nutrient intake, and physical activity are suggested as preventive measures to reduce risk of falls and fractures in the older population. Vitamin D is essential for upholding bone health and calcium retention, but older adults may have lower ability to manufacture vitamin D through the skin and are also often less exposed to sunshine (15). Thus, reduced muscle and bone mass may lead to increased morbidity. Amongst dietary requirements particularly important for this group, protein and vitamin D intake are specifically highlighted, as the intake levels required are higher for older adults than for younger adults (15, 16). Therefore, preparing for the body’s dietary needs in later years should start at an earlier age than most people realise.

Research also shows that older adults have less appetite and lower food intake than younger adults (17). Older adults may experience reduced appetite due to acute illnesses or chronic diseases (18). Most older adults use one or more medications to alleviate effects of different chronic conditions, which again can influence appetite negatively (19). Oral health deteriorates as an effect of ageing with poor dental health and reduced saliva production leading to chewing and swallowing problems (20). Other changes to the digestive system such as slower gastric emptying and changes in the microbiota and intestinal effectiveness also influence appetite (18, 21). Loss of appetite is the major cause of undernutrition in older people, with multiple potential underlying causes like decline in sensory perception, salivary dysfunction, poor oral health, various chronic conditions, psychological factors and polymedication (22). Food’s sensory and nutritional characteristics play an important role in food intake (5), and in older adults, it would be desirable to reduce the effects of satiation and satiety to allow greater energy and nutrient intake. Sensory properties are involved in food enjoyment and food desire, and an impairment in sensory perception in older age can mean a reduced appetite (18). In addition, as food is always consumed in a context (at home or away, alone or with others), the influence of eating context on food consumption and enjoyment is particularly important (23–27). Although energy intake is lower amongst older adults, nutrient requirements in this group are as high as for younger adults. This emphasises the need for understanding what can contribute to improve appetite and healthy food choice (28). More knowledge is needed to understand how older adults perceive and manage food in their daily lives and how the relation with food develops as old age advances.

Admitting that age is creeping up on oneself is not something that most people do. In a Norwegian study, this is elegantly explained by an informant: *‘Old age can come tomorrow’* (female, 84 years) (29). In affluent Western societies, 70-year-olds have more money, more freedom and better health than 70-year-olds had just a generation ago (30). However, there is no such thing as a typical older adult (31). Functionality can vary from being in very bad shape in the sixties to very good shape in the eighties (1). Despite this variability, the generally longer life expectancy means that laying the foundation for staying home and active for as long as possible is very important for both the individual and society (32). In addition to this, developing and marketing functional foods to older adults, which could potentially improve their nutritional status, is not always easy, as finding consistent segments of this target population is in itself a challenge: there are differences in needs and wants, cognitive status, demographics and life course, which, in turn, will affect the reasons underlying their food choices (33). Thus, it is very important to understand the factors that influence their food intake, to better tackle the risks associated to malnutrition (34, 35). Increased awareness amongst older adults and in the society of how ageing processes influence physical and mental performances is a central prerequisite to take preventive actions. For creating awareness, knowledge is necessary of home-living older adults’ food choice and food-related behaviour to plan for maintaining a healthy diet.

This study aims at better understanding what characterises food-related practices in active, home-living, self-reliant older adults, from retirement (67 years old) and well into older age (80+), in order to identify food-related factors that act as barriers and those that promote healthy ageing.

## Methods

Data collection methods and selection criteria were chosen to emphasise characteristics of home-living and self-reliant older adults. Selection criteria for respondents were based on the following reasoning:

1. Age from 67 years was chosen because this was the official retirement age in Norway at the time of the survey, and the change from active working life to retirement is a major life-changing event that influences daily routines and may lead to healthy as well as unhealthy food behaviours (36).
2. Equal distribution of men and women was desired, as investigating characteristics of the two genders was aimed at rather than how the two genders are represented in the population.
3. Ensuring participation of active home-living older adults was based on the assumption that the recruiting process itself predisposed selection of (active) home-living persons.

A three-pronged, mixed-methods approach was chosen to achieve this aim. First, a web-based quantitative survey was conducted to collect information about home-living older adults’ food choices and food-related behaviours in their daily lives (*N* = 1,005). Second, two focus groups with respondents aged 67–74 years (*N* = 7) and 75–84 years (*N* = 6), respectively, were conducted to shed light on aspects not adequately covered in, and questions raised by, the quantitative survey. Third, individual interviews (*N* = 10) provided in-depth insights on aspects of eating and enjoyment that were difficult to extract by other methods.

### Methodological and ethical considerations

#### Generalisability

The results from the study reflect the characteristics of the respondents as a healthy, active group and are not representative of the total population. Compared to the distribution in the population, examination of the survey sample showed that the age groups 67–69 years and 70–74 years were overrepresented in the study, whilst the oldest age group was underrepresented (Table 1). Furthermore, the requirement for an equal distribution of males and females caused the oldest age group to have a much larger male representation than the corresponding age segment in the population. Conclusions drawn from analyses of the data must be interpreted in light of the prerequisites of the data collection.

**Table 1:** Demographic characteristics of survey participants distributed into age groups (*n* = 1,005)

Demographic characteristics	Age groups				Total
	67–69 years	70–74 years	75–79 years	80+ years	
*N*	284	424	190	107	1,005
**Sex**					
-Female	59%	46%	53%	35%	50%
Distribution in the population*	58%	46%	54%	62%	55%
-Male	41%	54%	47%	65%	50%
Distribution in the population*	42%	54%	46%	38%	45%
**Education**					
-Primary school	6%	6%	6%	5%	6%
-High school	31%	31%	27%	37%	31%
-Univ <4 years	30%	33%	26%	21%	30%
-Univ >4 years	32%	30%	41%	36%	34%
**Household**					
-Living alone	25%	29%	43%	47%	32%
-Living together	75%	71%	57%	53%	68%

* Distribution of older adults in the Norwegian population based on sex and age (Available from: https://www.ssb.no/statbank/table/07459/tableViewLayout1/).

#### Ethics

The individual studies were planned and executed in accordance with the General Data Protection Regulation (GDPR) EU 2016/679. The principles of the Declaration of Helsinki 2008 for research on human subjects were the foundation on which the studies and data collection were constructed. The project was reviewed and approved by Nofima’s Ethical Board, and the studies comply with the Norwegian Data Protection Services code of conduct. All the information were voluntarily given by the participants both in the survey, as administered by the market agency, and in the qualitative studies, and a written informed consent was obtained in the qualitative studies. The details of the ethical considerations for each study are described below.

### Survey

#### Participants

A nationwide web-based survey amongst retired respondents aged 67 years and older was conducted in Norway in the fall of 2017. A Norwegian market agency, Norstat, was contacted to conduct the survey on behalf of the project. The respondents participating in the survey were drawn from Norstat’s consumer panel consisting of more than 81,000 respondents, where approximately 8,800 respondents were 67 years or older. Consumers in the panel have voluntarily signed up for participation and receive a small fee for their efforts. Participation in a study is voluntary, and recruitment continues until the desired quota for each requirement in a study protocol is reached. GDPR is observed by Norstat, and only anonymised data from the survey were delivered to the researchers. A total of 1,000 respondents were aimed at in this study. By using a web-based survey, the assumption was that the respondents being part of a web-panel were active and able and, thus, reflecting the target group of active home-living older adults. The recruitment was stopped after 1,005 respondents aged between 67 and 97 years had answered the questionnaire. The respondents were grouped into age groups: 67–69 years, 70–74 years, 75–79 years, and 80 years and older. Only 37 persons claimed not to participate in any activity, and thus we might assume that we reached the target group of active, older adults. Table 1 shows the distribution of subjects’ demographic characteristics.

#### Questionnaire

The questionnaire addressed the following food-related topics discussed in this paper:

- Meal routines (breakfast, lunch, dinner, evening meal, snacking between the main meals, eating with somebody else: yes/no)
- Food frequencies for main food items (several times a day, once a day, 3–6 times a week, 1–2 times a week, 2–3 times a month, once a month or less, never)
- Products they would change their consumption of (processed foods, meat, fish, fruits, vegetables [eat more, no change, eat less])
- Items important for food choice (sensory, preparation, packaging, labelling, impact on health; 1 = very important, 7 = not at all important) (Table 2)
- Use of nutrition information sources, use of medications (yes/no).

**Table 2:** Food choice items: ‘Please indicate how important each of the following items are for food choice on a scale from 1 = very important to 7 = not at all important’*

Items
1. The food looks appealing
2. Good taste
3. Like the food
4. The product is of high quality
5. International food with lots of flavour
6. Easy to chew
7. Easy to prepare
8. Easy to open packaging
9. The food can be stored in the package after opening
10. Small packages
11. Large letters on packaging
12. Cheap price
13. Well-known brand
14. Keyhole label
15. Natural product with few additives
16. Organic
17. Norwegian origin
18. Locally produced
19. Keep bodyweight stable
20. Increase bodyweight
21. Reduce bodyweight
22. Pay attention to one’s own health

* Items translated from Norwegian.

Three subscales from the health and taste scales were selected: general health interest, using food as a reward, and pleasure (1 = completely disagree; 7 = completely agree) (37). In addition, 24 food- and health-related statements were developed and included based on a group process amongst the partners in the project (Table 3). All items were measured on seven-point scales (1 = completely disagree to 7 = completely agree). Finally, demographics (Table 1), participation in activities, height, weight and satisfaction with life (1 = very unhappy to 10 = very happy) were measured. The questionnaire was pilot tested on persons in the relevant age group for timing, coherence and readability.

**Table 3:** Food and health statements: ‘Please indicate to what extent you disagree or agree with the following statements on a scale from 1 = completely disagree to 7 = completely agree’*

Statement
I am well acquainted with the government’s dietary advice for my age group.
I am quite healthy and have no special dietary needs.
I find it easy to eat enough food.
I am careful to drink enough during the day.
It is important for me to eat food with low salt content.
It is important for me to eat food high in fibre.
I know well the differences between different types of fat such as saturated/unsaturated fat.
It is important for me that my daily diet contains enough proteins.
I know that the protein requirement increases with age.
It is difficult to consume as much fruit and vegetables as required (five a day).
I would like to buy foods that are enriched with important nutrients.
I would like to buy foods labelled to reflect the nutrient requirements of my age group.
I eat smaller portions now than I did previously.
I don’t have as good an appetite as previously.
It is important for me to have good meal routines during the week.
I think it is cumbersome to prepare food.
I plan my meals so I can use leftovers later.
The price of food is not important; I buy what I want.
I like to challenge myself and try new foods.
I feel that the food has become monotonous and boring.
I don’t think the food tastes as good as before.
I wish I could eat together with others more often.
I wish I ate healthier.

* Items translated from Norwegian.

During planning of the study in May–June 2017, a check was performed for notification obligations to the Norwegian Data Protection Services. This did not trigger notification obligations. All the respondents have previously given their consent to participate in studies and be part of Norstat’s consumer panel.

### Focus groups

Based on the results of the survey, two focus groups were conducted in the summer of 2018 at Nofima to investigate in more detail the findings related to changes in food intake and factors that influenced the subjects’ current food routines. Nofima is a food research institute with facilities for conducting consumer tests and focus groups. To this end, Nofima has a database consisting mainly of contact persons to local clubs and organisations. Persons are recruited from this database to tests that for practical purposes need to be performed near or at the institute.

Topics from the survey that needed additional attention and were included in the focus group guide were food currently consumed and why, self-perceived changes in food choices and consumption, health issues of importance to food intake, strategies for food handling and what they missed or thought they might need regarding food (Table 4). Loss of abilities in older adults increase with age, and through a preliminary overview of the results from the survey, it was decided to differentiate the focus groups based on age below and above 80 years. In this way, we aimed at a discussion between respondents who should be on a similar level in their physical abilities as well as in life experiences.

**Table 4:** Focus group interview guide*

Questions
Introduction: Presentation of the background, interviewer, referent, and practicalities
Small talk: Tell me a little about yourself and the picture you sent us about your dinner dishes
Processed food: Attitudes towards ready-made meals – Are they natural? What do you think of when you read or hear about processed food?
Texture modified food: Can you name examples of some dinner products or dishes you no longer eat? Have you changed what you eat in terms of texture?
Packaging: What do you think about the packaging of the products you use most often? Do you have any strategi for open products that can be difficult to open?
Leftovers: When I say leftovers, what do you think? If you have leftover dishes, what do you do with them?
Future needs (meals and services): Would it make the everyday life easier for you if you had someone to help you with shopping and cooking? Home delivery service for food?

* Questions translated from Norwegian.

The first focus group consisted of seven older adults aged 67–76 years (see Table 5 for details). Recruitment was conducted through Nofima’s consumer database via e-mails to local organisations with older subjects. Recruitment criteria were that the subjects should be active, retired, living at home, and cooking their own meals. The participants each received a 300NOK (30€) gift card. The focus group was conducted during the daytime at Nofima’s facilities in Ås, outside of Oslo, Norway. In preparation for the first focus group, they were asked to send a picture of a dinner meal from the preceding week. All participation in the focus group was voluntary, and the participants were informed of their rights to withdraw at any given time before the start of the focus group. Participants recruited from Nofima’s database have voluntarily consented to be registered in the database for the purpose of participating in studies on food. Participants are informed that they can withdraw from the study at any given time without providing any explanation and that data they have contributed with, when possible, will be deleted from the data file.

**Table 5:** Focus group participants*

Respondent/sex	Age in years	Household	Family
**Focus group 1**			
Kristin: Female	67	-	Grandchildren
Mette: Female	69	Married	Grandchildren
Hanne: Female	70	Married	Grandchildren
Trine: Female	71	Married	Grandchildren
Raymond: Male	73	Cohabiting	Grandchildren
Birger: Male	74	Married	Grandchildren
Svein: Male	76	Married	Grandchildren
**Focus group 2**			
Nelly: Female	85	Living alone	Grandchildren, great-grandchildren
Ruth: Female	85	Living alone	Grandchildren, great-grandchildren
Gina: Female	85	Living alone	Grandchildren, great-grandchildren
Anette: Female	88	Living alone	Grandchildren, great-grandchildren
Beate: Female	89	Living alone	Grandchildren, great-grandchildren
Kari: Female	94	Living alone	Grandchildren, great-grandchildren

* Names of the participants are fictional.

The second focus group consisted of six older adults aged 85–94 years (see Table 5 for details). People belonging to this group are often not very mobile, and it was decided to conduct the second focus group at a senior centre during the daytime. Through acquaintances, an appointment was made with the manager at a senior centre in the eastern part of Oslo. Recruitment criteria were the same as for the first group: the subjects should be active, retired, living at home, and cooking their own meals. The manager recruited six participants, and the researchers were offered a room at the centre to conduct the focus group. The participants each received a 300NOK (30€) gift card. All the participants were informed of the study’s intention, so that they could withdraw their consent at any given time without providing any explanation, and that data they contributed with, when possible, would be removed from the study.

### In-depth interviews

Personal experiences are difficult to address in surveys or focus groups. To deepen our knowledge of what and why certain factors make food and meals enjoyable, 12 in-depth, semi-structured interviews were conducted in 2018. Two interviews were excluded due to a lack of responses from the interviewees, leaving 10 interviews for the final analysis (see Table 6 for details on participants).

**Table 6:** In-depth interview participants*

Respondent/sex	Age in years	Household
Martin: Male	69	Living alone
Albert: Male	69	Married
Alf: Male	74	Married
Emma: Female	75	Married
Odd: Male	75	Widower
Bente: Female	80	Married
Peder: Male	83	Married
Marte: Female	83	Married
Oda: Female	83	Living alone
Petra: Female	88	Widow

* Names of the participants are fictional.

The participants, aged 69–88 years, were contacted at cafeterias in shopping centres and during organised activities for older adults in the south-eastern part of Norway. Inclusion criteria were that they should have been retired for at least 2 years and did their own shopping and cooking. The respondents could choose where the interview should take place, and most of them invited the researcher home, whilst two interviews were conducted at a local voluntary centre.

The interview guide was structured over four main topics: what affects your food choices, how do you make a meal eaten alone pleasant, what influences your appetite, and in which situations do you most enjoy food (Table 7). Two pilot interviews were conducted to evaluate and improve the guide. This study was approved by the Norwegian Centre for Research Data (Ref. 57991).

**Table 7:** In-depth interview guide*

Questions
Introduction: Presentation of interviewer and practicalities
Small talk: Tell me a little about yourself
Food habits: Describe what a normal day looks like for you in terms of food (What kind of food do you usually eat to the different meals?)
Shopping: When you go to the store to buy food, is there anything in particular you look for in terms of packaging?
What do you think about the packaging of the products you use most often?
Appetite: In which situations are food most tempting?
Cooking: Which meal do you enjoy cooking the most?
Is there anything you particularly enjoy about cooking?
Is there anything you find boring about cooking?
What do you think about food from scratch compared to ready-made food?
Meals: What do your meals mean to you?
Which meal do you like best and why?
Can you describe the meal pattern you have now, and how it has developed over the past few years?
How do you plan your meals?
What is most important to you for a meal to taste good?
What makes a meal that you eat alone enjoyable/less enjoyable?
Does it depend on which meal it is?
Food pleasure: What do you think about the concept of food pleasure?
Can you describe your perfect meal as it is today with the resources you have available?
What is your dream meal?

* Questions translated from Norwegian.

### Data analysis

The construction of the web-survey was based on predefined hypotheses to select appropriate measurements for the generation of data. The guides for the focus groups and in-depth interviews were based on results from the survey where the data did not provide adequate information to answer the predefined hypotheses.

Data from the web-survey were analysed using SPSS (IBM SPSS Statistics vs. 26). Descriptive statistics were used to obtain an overview of the data. Pearson chi-square statistics were used to compare categorical variables. *T*-test was used to identify differences in means between groups. Factor analysis was used to identify patterns in the food behaviour statements.

The check all that apply questions (CATA) (meal frequency, physical activity and sources of information) were analysed with XLSTAT 2021.2.1 (Addinsoft, USA), using Cochran’s Q test, followed by multiple pairwise comparisons based on the McNemar–Bonferroni test, to identify significant differences amongst age groups for the different variables. Correspondence analysis (CA) based on the chi-square distance was conducted to visualise the contingency tables in two-dimensional plots.

Some of the attitude questions (e.g. food and health statements and food-related statements) as perceived by the different age groups were plotted via principal component analysis (PCA) (Pearson correlation) with XLSTAT 2021.2.1 (Addinsoft, USA). PCA is a multivariate reduction technique enabling the simultaneous analysis of the information provided by many variables, reducing the complexity into a reduced number of factors explaining a high percentage of the original information and allowing for visual comparison.

Data from the focus groups and interviews were recorded on an audio-recording device, transcribed and analysed using the software package ATLAS.ti (vs 6. ATLAS.ti Scientific Software Development).

## Results

### Survey

#### Food consumption

There were small differences between age groups regarding meal frequency. Over 95% reported eating breakfast every day, between 68% (80+ age group) and 77–81% (the other age groups) had lunch (*P* = 0.04), and approximately 98 and 50% consumed dinner and an evening meal, respectively. For the in-between meals, there was a slight increase from 5 to 11% (ns) in consumption from the youngest to oldest age groups for morning snack. Around 10% consumed a snack between lunch and dinner, whilst the after-dinner snack consumption was highest in the youngest (21%) and oldest age groups (19%) (ns), respectively. For both lunch and evening meal, the younger age groups were significantly more interested in eating together with somebody else (Table 8).

**Table 8:** Food and activity characteristics of the age groups (*N* = 1,005)

Variables	Age groups				Total	Chi-square	*p*
	67–69 years	70–74 years	75–79 years	80+ years			
*N*	284	424	190	107	1,005		
**Meals**							
-Breakfast	95%	97%	96%	97%	96%	2.381	ns
Eat with someone	66%	64%	56%	55%	62%	8.466	ns
-Snack between breakfast/lunch	5%	5%	8%	11%	6%	6.049	ns
-Lunch	81%	77%	81%	68%	78%	8.302	0.04
Eat with someone	85%	79%	77%	65%	79%	22.965	0.001
-Snack between lunch/dinner	11%	10%	11%	7%	10%	1.051	ns
-Dinner	98%	98%	99%	96%	98%	2.944	ns
Eat with someone	92%	90%	87%	83%	89%	10.642	ns
-Snack between dinner/evening meal	21%	14%	14%	19%	17%	7.571	0.056
-Evening meal	48%	53%	53%	47%	51%	2.865	ns
Eat with someone	62%	59%	47%	47%	56%	17.271	0.008
**Foods consumed 1–2 t/week or more often**							
-Milk	60%	55%	61%	61%	58%	2.849	ns
-Red fish	39%	48%	44%	40%	44%	5.951	ns
-White fish	54%	58%	61%	69%	58%	8.260	0.04
-Fruits	82%	88%	90%	91%	87%	8.857	0.03
-Vegetables	86%	88%	83%	85%	86%	3.062	ns
-Beef	22%	22%	23%	14%	21%	3.924	ns
-Pork	25%	26%	28%	21%	26%	1.595	ns
-Chicken	34%	30%	37%	27%	32%	4.428	ns
-Meatballs	22%	23%	29%	38%	25%	13.735	0.003
-Ready-to-eat meals	5%	4%	7%	15%	6%	22.114	<0.001
-Dry food	4%	7%	8%	11%	7%	7.953	0.05
**Activities**							
-Fitness centres	29%	29%	31%	24%	29%	1.531	ns
-Go for a walk	87%	89%	82%	69%	85%	29.854	<0.001
-Participate in social group/club	43%	46%	50%	50%	46%	3.056	ns
-Senior centre	3%	5%	15%	31%	9%	91.654	<0.001
-Other	20%	26%	26%	29%	25%	5.101	ns
**Health**							
-More fit	26%	28%	31%	40%	30%		
-Same as others	50%	53%	54%	50%	52%		
-Less fit	23%	19%	15%	9%	18%	15.214	0.02
Use medications regularly (yes)	77%	81%	77%	86%	79%	5.215	ns

Foods and drinks most frequently consumed were dairy products (milk, fermented milk drinks, yoghurt, semi-solid yellow cheese and Norwegian brown cheese), bread, crisp bread, cereals, eggs, meat and fish spreads, fruits and berries, water, coffee and tea. There were no statistically significant differences in consumption frequencies between age groups except for fish spreads, which were consumed more often by the 80+ group (*P* = 0.04). Products normally used for dinner that were most frequently consumed (1–2 times a week and more) were white fish, red fish, chicken, pork meat, meatballs, and beef meat, in that order. The 80+ age group consumed fruits (*P* = 0.03), white fish (*P* = 0.04) and meatballs (*P* = 0.003) more often than the other age groups (Table 8). The respondents indicated that they would like to eat more fish (57%), vegetables (50%) and fruits (47%) and eat less processed foods (30%) and meat (17%). There were no systematic differences across age groups.

**Fig. 1:**
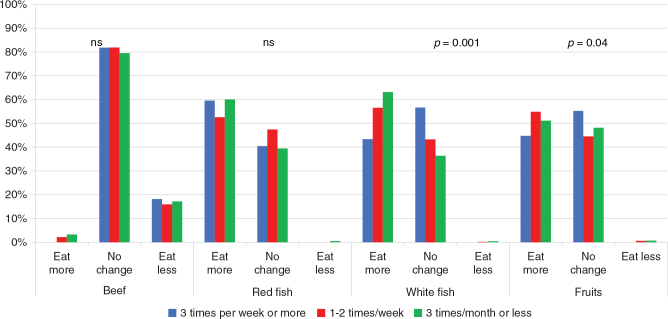
Intention to change consumption for beef, red fish, white fish, and fruits distributed on reported frequency of consumption. Percentages within each food category (*N* = 1,005).

To compare ‘intention to change consumption of’ categories with reported intake of relevant foods, food frequencies for beef, pork, chicken, meatballs, red fish, white fish, fruits, vegetables, ready-to-eat meals, and dried foods were recoded into three or more times per week, 1–2 times per week, and three times per month or less. For both white fish (chi-square 18.978, *P* = 0.001) and fruits (chi-square 11.681, *P* = 0.02), the intention to increase consumption corresponded with reported low intakes (Fig. 1). For red fish, beef, pork, chicken, vegetables, and processed foods, intention to change consumption was independent of reported consumption.

#### Activity and health status

**Fig. 2:**
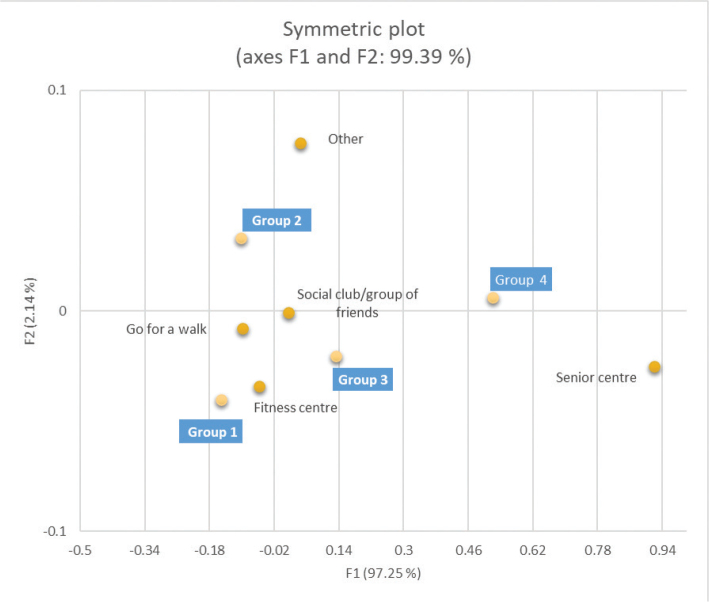
Representation of the activities reported by different age groups in the first and second dimensions of the correspondence analysis. Group 1: 67–69 years, Group 2: 70–74 years, Group 3: 75–79 years, Group 4: 80+ years.

In the study population, only 3.7% reported not participating in any activity. The activity most frequently selected in all age groups was going for a walk, decreasing from 89 to 69% in the oldest group (*p* < 0.001). The age group 80+ years more frequently went to senior centres (31%) compared to the younger age groups (*p* < 0.001) (Table 5, Fig. 2).

On the question ‘How fit are you compared to others your age?’, the 80+ age group scored significantly higher than the younger age groups in thinking they were more fit (*P* = 0.019). The General Health Interest and Reward scales had good reliability scores (Cronbach’s α = 0.745 and 0.741, respectively), whilst the Pleasure scale did not (α = 0.560 with the item ‘I eat the food even if I don’t like it’ removed). All the age groups thought the topics were important.

#### Food-related statements

Almost one- quarter of the respondents agreed (score 5–7) with the statement ‘I feel that food preparation is a burden to me’. The statement ‘I wish I ate healthier’ was recoded into ‘Healthy eating’ (54%, score 1–4) and ‘Not eating healthy enough’ (46%, score 5–7). There was a significant difference between the groups who thought they ate healthy (mean = 7.99) and those who thought they did not (mean = 7.60) in their perceived satisfaction with life (*t*-test *F* 8.823, *P* = 0.003). Similarly, there was a significant difference in Body Mass Index (BMI) between these groups (*t*-test, *F* 11.339, *P* = 0.001). Those who thought they ate healthy had a lower BMI (mean 25.3 ± 3.5) versus those who reported not to eat so healthy (mean 26.6 ± 4.3). No difference was found for sex, age group, or education.

**Fig. 3:**
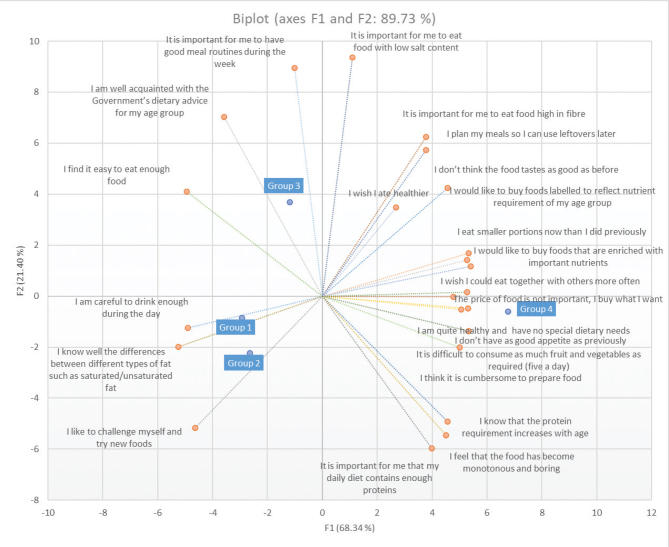
Importance of food and health statements for different age groups (from Table 3). First two components of the principal component analysis account for 89.7% of the variability. Group 1: 67–69 years, Group 2: 70–74 years, Group 3: 75–79 years, Group 4: 80+ years.

A general linear model (GLM) showed that the 80+ age group scored significantly higher than the other age groups for the items *Difficult to eat five times a day* (*F* = 3.545, *P* = 0.01), *Eat smaller portions* (*F* = 3.938, *P* = 0.008), *Lower appetite* (*F* = 5.413, *P* = 0.001) and *Less tasty* (*F* = 2.927, *P* = 0.03). Figure 3 displays the multivariate representation, via PCA, of all the food and health statements (Table 3), as related to the four age groups. The younger age groups, 1 to 3, were more confident regarding nutritional advice and gave importance to more general routines like drinking water and having good meal routines. They were confident about their nutrition (‘I find it easy to eat enough food’) and thought trying new foods was important. Meanwhile, group 4 was more linked to the barriers that may arise with ageing: they were more concerned about preparation difficulties (‘I think it is cumbersome to prepare food’), sensory losses and lack of appetite (‘I don’t think the food tastes as good as before’; ‘I feel that the food has become monotonous and boring’) and lack of social interactions (‘I wish I could eat together with others more often’). Furthermore, nutritional concerns for group 4 were focused on specific nutrients (proteins and fibre) and a realisation that they may not be having enough food (‘I eat smaller portions now than I did previously’), particularly for some food groups (‘It is difficult to consume as much fruit and vegetables as required [five a day]’).

**Fig. 4:**
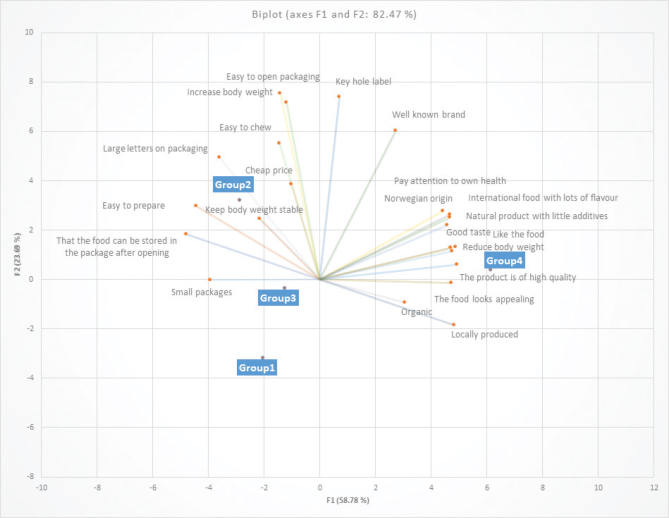
Importance of food-related statements in different age groups. Two first components of the principal component analysis, accounting for 82.5% of the variability. Group 1: 67–69 years, Group 2: 70–74 years, Group 3: 75–79 years, Group 4: 80+ years.

The items the respondents thought were most important for food choice (Table 2) were related to sensory aspects of the food (items 1–4, mean 1.73–2.41) and *Natural product* (mean 2.65 SD ± 1.54), whilst *Increasing weight* was least important (mean 5.97 SD ± 1.81). Comparing food choice items by age group by general linear modelling showed statistically significant differences between the age groups for *Appetizing* (*P* = 0.03), *Quality* (*P* = 0.04), *Small packages* (*P* = 0.05), *Reduce weight* (*P* = 0.003) and *Health consequences* (*P* = 0.008) (see also Fig. 4). For all items, the youngest age group thought the items were more important than the oldest did, except for the *Small package* item, which the oldest group thought was the most important. The *Price* item (*P* = 0.04) also differed between age groups, although not in a systematic direction. Figure 4 displays the multivariate representation, via PCA, of reasons underlying food choices for the four age groups, as displayed in Table 2.

**Fig. 5:**
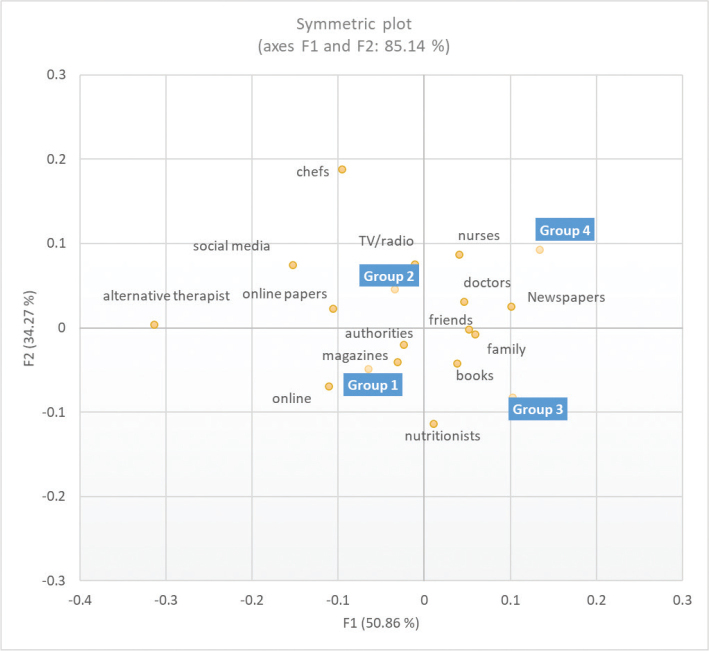
Representation of the information sources mostly used by different age groups in the first and second dimensions of the correspondence analysis. Group 1: 67–69 years, Group 2: 70–74 years, Group 3: 75–79 years, Group 4: 80+ years.

The respondents were also asked to indicate where they got information about food and health (CATA question). Older age groups (3 and 4) most often selected traditional sources of information, such as health personnel and newspapers, friends and family, whilst the youngest age groups choose more frequently online resources like social media and online papers, as well as chefs and alternative therapists (Fig. 5).

### Focus groups

Findings from the survey showed that the oldest age group, 80+ years, differed from the younger age groups. The survey did not provide information about why the respondents chose the different foods, or whether or why food habits had changed over time. Two focus groups with respondents under and over 80 years, respectively, were conducted to elicit more information about the following topics: food consumption and changes, health, and strategies for food choice and behaviour.

#### Food consumption

In the youngest focus group, all participants who had brought a picture showing a dinner meal showed dishes consisting of fish. In the oldest group, all the respondents said they mostly ate fish or fish products. In addition, they also consumed chicken and, quite frequently, ready-to-eat meals. In more detail, the younger respondents stressed the importance of cooking meals from scratch. They tried not to eat processed foods, but they were unclear about what could be termed processed. ‘*At the store they sell salmon burger, and it’s just minced salmon, nothing added; you just get it like a burger. That’s processed with nothing added, so I think it’s not processed, but still processed*’ (Hanne, 70). ‘*Processed foods we know often contain much salt and maybe also much fat, but we still eat sausages quite frequently – it’s very convenient*’ (Svein, 76). The younger respondents did report having a good appetite, although they did eat smaller portions as described by Raymond (73): *‘I remember before when we had pork chops, for instance. Then I always had two and at least 2–3 potatoes. Now I think how on earth did I manage to eat two chops for dinner*’*.* Cooking their own food was not considered to be a problem. ‘*We’re used to buying all the ingredients we need … it’s a little strange to buy such (ready-to-eat-meals) now; I have a barrier to that*’ (Raymond, 73). The older respondents were frequent users of the senior centre and sometimes ordered dinner. ‘*I have ordered dinner today. One gets used to eating early*’ (Kari, 94).

#### Food and health

Several respondents mentioned that dishes not consumed anymore were typical traditional dishes often with smoked meat and containing much salt. Both groups commented that dishes with offal were not used, and some of the respondents said it had something to do with health. One man commented that he ate healthier than previously: ‘*One thinks a bit more about health than before. One sees that when one has had (medical issues), then you get some messages you have to follow*’ (Raymond, 73). Another man said, ‘*When we get to our age, we get defects and illnesses and such, and then you get dietary advice…. from doctors or brochures*’ (Svein, 76). In the older group, lack of taste and appetite was noted: ‘*It’s not the same taste when one lives alone*’ (Beate, 89). ‘*To be quite honest, I eat because I have to eat. It’s like nothing interests me anymore. It’s like… I’ve heard about many who don’t eat. No, I’m never hungry, but I eat every third hour when I’m home*’ (Beate, 89). Several others also agreed that they had regularly timed meals, but it was not clear whether this was their own idea or whether they had been told to. ‘*I don’t understand what happens when one gets to be so old. I have become another person after becoming so old*’ (Kari, 94). One woman commented that all the fruits, vegetables and breads one was advised to eat made her stomach feel inflated, which was very uncomfortable. She said the only solution for this ‘*was to eat white bread and minced meat (laughter)*’ (Mette, 69).

#### Food behaviour strategies

None of the respondents wasted food. ‘*The green bag is almost always empty* [authors’ comment: the green bag is for recycling food waste]’ (Kari, 94). Leftovers were reused either on their own, as an ingredient in a new dish, or frozen for later use. They commented that they had been taught as children not to waste food; they just removed mouldy parts of bread and jam and ate the rest. They further mentioned that all their children were concerned with ‘best before’ labelling, which they were not. Packaging issues were important to the group. They all agreed that they wanted to see the product to assess the quality, particularly fresh products like meat, fish, fruits and vegetables. Opening the packages was solved by using nutcrackers, rubber gloves, knives or scissors, but it did not come out as a reason for avoiding a product. ‘*One often buys the same product, and then one knows how to open it. I think sometimes it’s hard to find where it says open here … so then I just use a knife or scissors. That is simple, and it works really well*’ (Svein, 76). The respondents were concerned for ‘*those who really struggle, with rheumatism and such, that it’s not easy*’ (Kristin, 71).

### In-depth interviews

From both the survey and the focus groups, particularly amonst the oldest participants, lower enjoyment of eating came up as an obstacle to consuming enough food. To investigate which parameters older adults associated with pleasure of eating, and thus increased food intake, 10 in-depth interviews were conducted. Food choice, appetite, strategy for making a meal pleasant and eating context were investigated.

#### Food choice

Health concerns were important for several of the informants in their food choices. For instance, a couple said they had changed from using white bread to coarse bread, and in porridges, they changed from rice to a combination of rice and whole wheat. ‘*We are very conscious not to buy white bread, not even to shrimps* [authors’ comment: shrimp open sandwich with white bread is a classic Norwegian combination]’ (Marte, 83). Fish was also mentioned by the informants. One woman said that she was really fond of salmon and reflected that the liking might also be due to her knowledge that it was healthy: ‘*Maybe it plays a role that you know it is healthy. It is probably in the back of my head; I think so*’ (Bente, 80).

One informant said he could not cook, and, except for breakfast, he used ready-to-eat meals. The only meal he liked to cook was breakfast with eggs and bacon, because he knew how to do. Ready-to-eat meals were perceived by the informants to be convenient, but tasteless, and not so healthy as meals made from scratch. Still, these meals were used frequently.

One informant complained that foods were often only available in big packages and that she sometimes even had to throw away the food. ‘*I am actually surprised there are so few things one can buy in small packages, because we are many old people*’ (Petra, 88). Another respondent said he liked to buy big packages, partition and freeze. ‘*I buy big, to put it like that. If it is frozen, it goes straight into the freezer, and if it is fresh, one can portion it*’ (Martin, 69).

#### Appetite

Taste and smell were mentioned by some of the informants as being important for both appetite and enjoyment of the food. For others, particularly for those who did not think so much about food or thought food was just something you had to have, taste and smell were less important. Similarly, how the food was presented on the plate was important for some to increase appetite, whilst not for others. ‘*If the food tastes good, I don’t mind if it’s messy on the plate or the serving tray*’ (Albert, 69).

Hunger was a driver for appetite for some of the respondents whilst not for others: ‘*It is often that I just look at the watch*’ (Martin, 69). Many mentioned that they were most hungry in the morning and that their appetite decreased the later it was in the day. Appetite was also influenced if the meal was considered a pleasure or duty. ‘*The dinner is totally uninteresting, and it is just a duty*’ (Petra, 88). ‘*It is just like a burden to me. That I have to eat right? To survive*’ (Oda, 75).

### Strategy for making a meal pleasant

Dinner was the meal that was most problematic for the informants, as this meal most often was or had been consumed in company with others. ‘*When you are two, that is the right thing; then I enjoy the food more than when I sit and eat the food all alone*’ (Albert, 69). Another informant who lived alone said, ‘*And then I set the table with a tablecloth, napkins, and salt and pepper and everything. Just as usual (when her husband lived). I do that also now when eating is a duty*’ (Petra, 88). One informant pointed out that light was important for food enjoyment and influenced taste: ‘*In general, I want good light when I’m eating. I like to see the food, and if it’s fish, I like to see if it’s got bones in it*’ (Albert, 69).

### Eating context

The type of food consumed, or the taste of the food, was not the sole reason for whether a meal was enjoyable. One woman stated that her most enjoyable meal was with the closest family under perfect conditions abroad. She could not remember at all what they had eaten. ‘*I think that the people around maybe mean much, much more than what I eat*’ (Bente, 80). The informants were almost unanimous in that food eaten in company was more enjoyable than solitary eating, although taste was not always the most important factor. ‘*The food by itself doesn’t taste better, but I think that sitting with someone makes you enjoy the meal more than you do when you just push it in to finish*’ (Martin, 69).

Dinner was the meal in which eating alone was most problematic. However, the company one had should be good friends or family, and not too many. ‘*If it is too many, then it can, that is, it can be too much noise around the eating situation*’ (Alf, 74). ‘*If you meet four people you have never talked with, and you shall sit by the table and enjoy the food…. It is a little uncomfortable for me then*’ (Albert, 69).

The informants had different views on what they liked, but a common denominator was that they knew they had to eat and eat healthy. Furthermore, the social aspect of eating was particularly important, as some of them struggled with less hunger and appetite, or they were more alone in their daily life.

## Discussion

Most previous studies on malnutrition in the elderly have focused on the treatment of different pathologies once established, rather than looking into prevention by targeting the underlying determinants (22). As highlighted by Maitre et al., malnutrition in the older ages has scarcely been investigated in relation to food attitudes, preferences and habits (38). In the present study, we contribute to enlarge the knowledge in that area, investigating food-related factors and practices amongst active and home-living older adults. Such knowledge may contribute to implementing strategies for better eating amongst less able older adults, as a main challenge for many is to eat enough to maintain good health and stay healthy longer, avoiding or delaying negative consequences of malnutrition. Reasons underlying malnutrition are complex and varied, and they interact, including nutritional and non-nutritional issues like physical function and health problems as well as cognitive, affective and sensory functions (39).

The results from the present study highlight how increasing age impacts older adults’ food perception and how they organise their food-related behaviours. Although there is large between-individual variability in older adults’ capacities (5), a noticeable change was evident from around 80 years of age. An overall finding was that the oldest age group, 80+ years, was more concerned with perceived difficulties and barriers than the younger age groups. This is in line with findings from another study (38). In their study on nutritional status and food-related practices amongst French older adults, Maitre et al. (38) identified two clusters of consumers mainly differentiated by age below and above 80 years. The findings from our study will therefore primarily be discussed within this perspective.

### Food consumption

Good and nutritious food is essential for older adults to maintain good health. The food intakes reported by the active older adults in our study showed that they had a diet that was very much in line with dietary recommendations (16). They consumed a varied diet and, in particular, reported high consumption of fish, fruits and vegetables. This is also consistent with other findings from Norway (40, 41). Although our study did not collect data from other age groups, statistics from the Norwegian survey on living conditions (42) confirm that older adults report high consumption of fish, fruits and vegetables and higher than younger age groups.

The younger focus group (67–76 years) in our study highlighted fish consumption, as all the dinner meal photos they provided for the discussion featured fish. This might be an artefact caused by a desire to show how healthy their meals were (social-desirability bias), as no instructions were given about a particular day the photo should be taken. Still, the discussion prompted by the pictures showed knowledge about what a healthy meal should be as well as actual food consumed. In the older focus group (85–94 years), the discussion started by agreeing that they mostly consumed fish before they mentioned other food items such as chicken, along the same line as the younger group, highlighting the importance of fish in their diets. This is in agreement with the results from the quantitative part of our study (survey) where respondents 80+ years also reported eating more white fish as compared to the younger group. Traditionally in Norway, white fish consumption was most common until fish farming made red fish more available (43). This shift occurred around the 1980s, which might account for the differences in fish choices between the age groups, with the older group having been brought up more exposed to white fish usage.

Whitelock and Ensaff found that changes in food consumption, such as eating less red meat and more fish, were attributed to oral problems (44). In our study, this was not reported by the respondents, although it was explicitly explored by the moderator in the focus groups. One reason might be that our respondents were selected to belong to a group with good health and thus possibly more resources, for instance with access to regular dental care (45), whilst respondents in Whitelock and Ensaff’s (44) study belonged to a group with multiple deprivations. Another reason might be that the respondents did not think of oral problems in relation to changes in their consumption practises, although they did report frequent consumption of minced meat products such as sausages and meatballs and less consumption of whole meat products. Such changes might have been experienced slowly, with older adults resourcing to softer, easier-to-chew products to compensate for a loss in chewing capability, without a conscious realisation of the reason. When texture modification is discussed in relation to elderly product development, usually it is targeted to a population with certain diagnosed issues in chewing or swallowing, dysphagia, or needing special nutrition (46). However, some literature focusing on sensory reasons underlying food enjoyment and rejection in the elderly, with a focus on oral processing and oral comfort, shows that the complexity of the issue goes further than soft/hard textures (47), and product-related attributes like dryness can be responsible for food avoidance rather than oral health. Further research on this topic is needed, as this might contribute to targeted product development as well as nutrition advice to older adults.

### Appetite

Results from the survey showed that the respondents in general reported having a good appetite, but they ate less than before. An interesting finding was the surprise shown in the younger focus group when discussing current food consumption as compared to their eating habits whilst in active working life. In this focus group, participants did not report a problem with appetite; however, in the discussion, they agreed that they had reduced their food intake considerably from their working years. Previous research postulates that motivation to eat may be reduced with increasing age, even though liking is not diminished (5). The apparent lack of awareness amongst the respondents in the younger focus group about how or why they had reduced their food intake can, in a larger picture, be a nutritional challenge, as older adults may not compensate reduced food intake with more nutrient-dense foods due to changed dietary requirements.

For the respondents 80 years and older, more difficulties were mentioned, both in how much they were able to eat and in terms of lack of appetite. Our findings are in line with several studies that show how reduced appetite is common amongst older persons and increases with advancing age (18, 48–50), increasing the risk of malnutrition also in home-living elderly (51). Loss of a partner and/or friends becomes more common the older one gets, which again leads to more time spent alone (27). Loneliness is an important factor influencing appetite and quality of the diet negatively (27). Our survey showed that a majority of the respondents wished to eat meals with others, although this wish was slightly reduced with age. This could imply that loneliness was somewhat accepted with increasing age, or that other factors such as feeling more tired played a larger role. However, the following in-depth explorations from the interview study expanded on this finding and showed that for the oldest respondents, loneliness, such as through loss of partner, was a major reason for lack of appetite and enjoyment of food. This is confirmed by others who find that lack of appetite is closely associated with depression and loneliness (38, 52–54). On the other hand, most respondents said that being with others, particularly family or friends, increased their appetite and food enjoyment. Our study further confirmed findings that whilst not all communal eating was positive (27, 55), eating together with somebody you like was a sure ingredient to an enjoyable meal. Previous studies with older adults in Norway also suggested that a social network and social eating were important for this age group and that loneliness was one of the things older adults were most afraid of (56). In addition, research shows that good nutritional status is strongly connected with enjoyment and pleasure amongst older adults (53). How to maintain and promote meal socialisation amongst the elderly is a challenge to be solved by Western societies, as loneliness is a recognised risk factor for malnutrition (57) that needs to be addressed.

### Food and health

Medicine use is one factor influencing appetite (44). In our study, despite high reported use of medications across all age groups in the quantitative study, no significant difference in use of medications could be seen between the age groups. As our study focused on active and self-sufficient older adults, medication therefore might not be a prominent factor influencing appetite in this consumer segment.

To maintain good appetite and, consequently, facilitate a nutritionally acceptable diet, good taste is of major importance. Our findings showed that this factor needs be taken into account in planning a healthy diet: taste and liking scored highest on importance of all food characteristics. Similar findings have been shown in other studies in the adult population (58) and older adults living at home (59). Whitelock and Ensaff found that older adults’ food choices often were based on sensory aspects as well as enjoyment, to the exclusion of other factors (44). An important consideration is, therefore, that sensory decline for both taste and olfaction has been found in older adults (5, 49, 60), although changes occur so slowly that this might not be acknowledged by the respondents. However, some of the older consumers in our study reported that food did not taste so good or matter so much any longer. We cannot say whether this is a result of sensory losses or other underlying reasons, as older consumers often report that food no longer tastes good because of psychological or physiological issues such as grief, mood changes and lack of outdoor activities (59, 61). In both circumstances of decline in taste perception and food liking, the implications are that attention to sensory attributes and preference is particularly important in the development of healthy food products for older age groups.

In our research study, comments indicated that the respondents were aware of the health consequences of not eating enough and that having daily meal routines helped to keep up food intake. Routines can be good, particularly for those living on their own (55). Previous studies showed that older women living alone simplified cooking and eating practises, and that elderly men with poor cooking skills had challenges to improve their energy intake (25). Our studies showed gender differences in how they organised their food. This is also confirmed by others (62, 63). Being able to buy smaller packages was also mentioned by the respondents. Although many declared the preference for cooking from scratch, buying smaller meals was also an option, which is in line with the findings of others (64). Partitioning meals into smaller portions, either for consuming later or for freezing, seemed to be a strategy amongst the respondents in this study. Furthermore, making smaller portions for later use was considered a convenient way not to cook from scratch every day, as well as to avoid wasting food. This corresponds with earlier findings (7) and seems to be one strategy for being able to consume a whole meal amongst small eaters with little appetite. However, making and consuming smaller portions may also be a risk for undernutrition if the smaller portions are not compensated with more eating episodes (65).

The characteristic identified in this study that personal resources were important for maintaining a good diet was confirmed in other studies (55, 61, 66), which showed that higher personal resilience was positive for promoting health in older age. Despite most of the respondents reporting a sound nutritional practise, the respondents displayed a wish to eat healthier. In the survey, this was particularly evident amongst consumers who reported lower consumption of white fish and fruits.

## Conclusions and future work

This study has used a mixed-methods approach to provide insights into the behaviours and needs in a population group of active, home-living older adults.

We have seen clear differences in food-related behaviour between the younger and older adults of this group, which emphasises the need to differentiate measures that may uphold a healthy diet and lifestyle. Whilst the younger age group, 67–79 years, perceived their daily life to be a continuation of working life, although with reduced food intake, the oldest age group, over 80 years, experienced more barriers impacting their daily diet.

The respondents in our study referred to several strategies contributing to maintain daily food intake. Intention to eat healthy and adherence to dietary advice were prominent amongst the active home-living older adults. Eating with others and a convivial social setting were important factors for maintaining or increasing food intake. However, putting this into practice needs further study, as it requires different solutions depending on personal as well as local characteristics.

Providing healthy foods and fortified foods, if necessary, that fit into older adults’ daily routines needs to be further explored as a means to improve food intake amongst older adults for preventing malnutrition.

A gender difference was noted. Women more often were tired of preparing food, whilst men lacked the knowledge to do so. Devising and implementing strategies for preventive purposes to prepare older persons for the changes in their daily food preparation routines, particularly after retirement, may ensure better food intake in their later years.

## Limitations

This study focused on food-related behaviour in an active and well-functioning segment of older adults and did not aim for a characterisation of the general older population. Thus, it is not possible to determine the extent of food-related problems in the general older population based on our research. However, our findings highlight insights that can moderate the challenges facing older adults and that are described in other studies (44, 49, 67). We also did not collect information about economic resources or living arrangements, which have been found to influence healthy eating in older adults (68); less well-situated persons are more vulnerable to unhealthy diets.

The three studies described in this paper were conducted in 2017 and 2018, and findings and conclusions from the studies might be subject to changes over time. For instance, the COVID pandemic in 2020 and 2021 would most probably have influenced the results in that many older adults were highly affected by the imposed isolation and because of their particular vulnerability. We can hypothesise that the loneliness aspect and need for social intercourse would be even more pronounced. However, the results do not reflect specific societal events and correspond well with what others have found over a longer time span. Therefore, we uphold that the findings provide valuable and valid insights into active older adults’ food-related behaviours and what is desirable for a good life.
